# COVID-19 Deaths Cases Impact on Oil Prices: Probable Scenarios on Saudi Arabia Economy

**DOI:** 10.3389/fpubh.2021.620875

**Published:** 2021-02-04

**Authors:** Abdelmageed Algamdi, Said Khalfa Mokhtar Brika, Adam Musa, Khalil Chergui

**Affiliations:** ^1^Department of Computer and Information Systems, Community College, University of Bisha, Bisha, Saudi Arabia; ^2^Department of Administrative Sciences, Community College, University of Bisha, Bisha, Saudi Arabia; ^3^Department of Administrative Sciences, Community College, University of Bisha, Bisha, Saudi Arabia; ^4^Department of Management Sciences, Faculty of Economics, University of Oum El Bouaghi, Oum El Bouaghi, Algeria

**Keywords:** COVID-19, oil prices, epidemic, ARDL, Saudi Arabia (KSA)

## Abstract

The purpose of this paper is to discuss death cases on the World, exacerbated investor fears, uncertainties, and increased volatility of crude oil prices in financial markets. The reaction absorbed the epidemic gradually until January 22. Still, the market situation changed soon with a sharp drop in prices, and prices slowly recovered after that until June 14. The data of this research using an econometric model, the ARDL (Autoregressive Distributed Lag), according to the Gets methodology, using daily data, January 22 –June 14, 2020. Our ARDL shows, the death ratio has a significant negative effect on oil price dynamics. However, the death ratio has an indirect impact on volatility in Crude Oil prices. The findings show that the death toll of COVID-19 has a significant impact on oil prices in Saudi Arabia (KSA). However, the preliminary results mainly influence by the situation reported in the USA. When we assess the case outside the USA, and we see the positive effect of the COVID-19 death figures on oil prices, therefore, stress the amplification of death-related risks to the financial market and the real economy, caused by increased, policy-induced economic uncertainty in the United States.

## Introduction

In Saudi Arabia, the oil industry is essential and has significant spills over into certain services industries like travel, Business, IT, and religious tourism. The slump in oil prices will not have a substantial impact on export earnings, as a favorable regulatory framework drives oil production stability. Since oil prices are an exact driver of inflation, the fall in oil prices will have a positive effect on the consumer price index, and the most significant impact of the shock in oil prices will be the decline in investment. Covid-19 economic disorder led to substantial decreases in energy demand and prices, particularly of oil and industrial metals (1). Declines in commodity prices might interfere with financial uncertainty and investments and growth in producing and exporting oil and metals countries. Still, they could be beneficial for importers, business users, and consumers (2).

Believed to be the downturn in general economic activity and transport in particular, which accounts for two-thirds of world oil demand, has seriously affected oil markets. Between January and April, oil prices declined by two thirds with a record sharp 1 month decline compounded by the OPEC-Partnership Deal breakdown leading to a supply slump. Oil exporters are affected by falling prices, and their economies are expecting to sink by 4.4% in 2020 (3).

**Figure 1:**
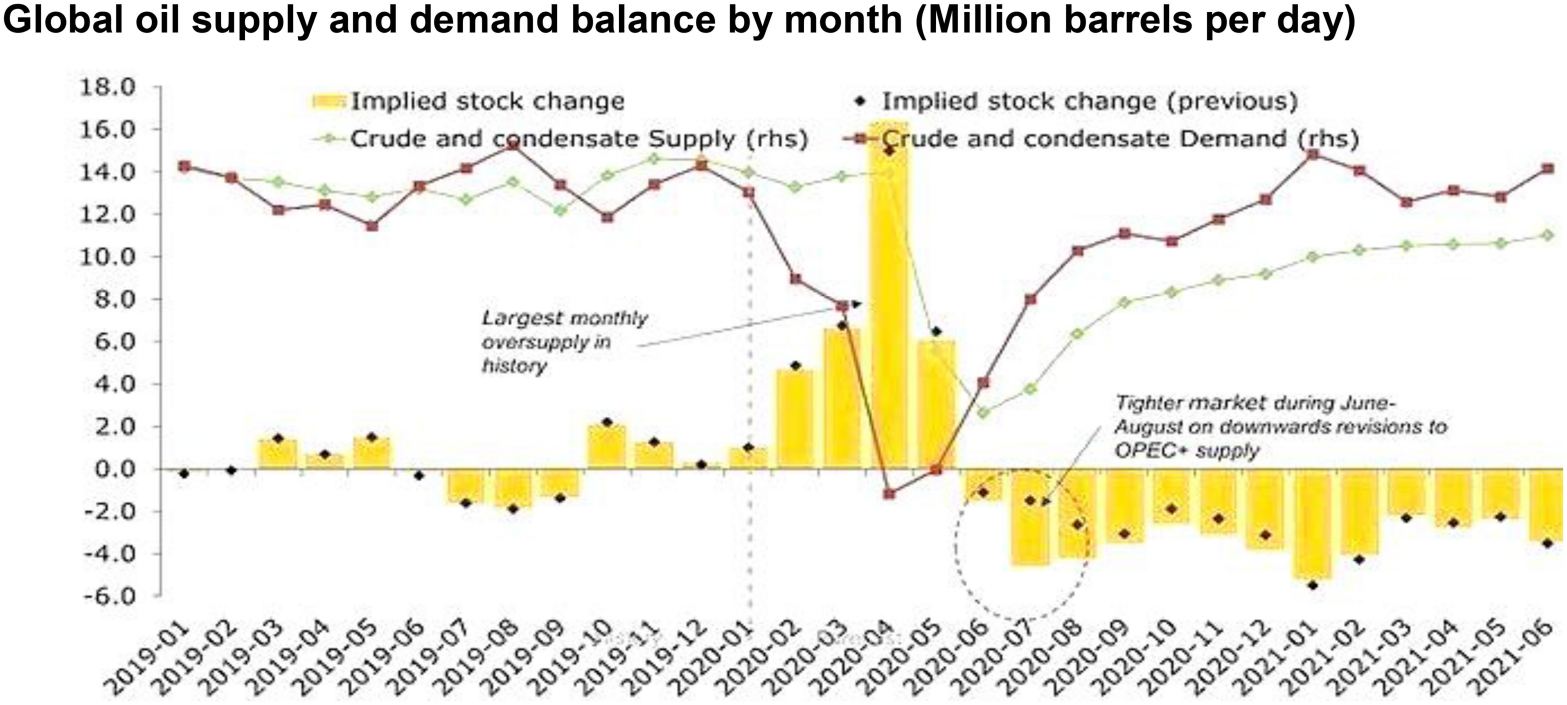
Global oil supply and demand balance by month (Million barrels per day). Source: (4).

It is well-known that the historical and predictive routes of global oil demand and economic growth have been outlined in Figure 1. There is a quick "V” shaped economic recovery in the second half of 2020 under the single-wave scenario, but oil demand does not fully recover from the prepandemic trend. The pessimistic scenario is that oil demand hits 2019 peaks only and remains far below the pre-pandemic rate until 2023. The latest pandemic progress indicates that some countries that an outcome similar to this catastrophic scenario is more probable.

Generally accepted in the long run, new models of service, replacement of video conferencing for transportation, continuing travel constraints, and individual travel resistance may lead to an ongoing reduction in demand for travel and fuel (5). Pressures on global supply chains, re-shore production, and domestic import substitution may also reduce the demand (6).

Until now, the Kingdom of Saudi Arabia, one of the countries affected by the Coronavirus outbreak, has taken steps to slow the spread of the pandemic with a growing number of cases, and governments are likely to face economic repercussions as the company slows down. People and wealth movement and investment, but the epidemic has made the collapse of stock markets a reality in all parts of the World (7, 8), and will cause damage (9, 10). The economic outlook was expected, at least in the short term, which cannot be avoided. Many countries around the World are in a recession (11, 12). The negative consequences expected to worsen, and lower oil prices may lead to additional financial pressures on the economy.

## Literature Review

The OPEC+ member strategy to reduce supply to support price recovery now focusses aggressively not only on stocks but also on the shape of the oil curve and the nature of an approach to supporting the short-term market (8, 13, 14). spot prices are higher than futures contracts, allowing refiners and traders to sell off oil inventories (15).

(16) has provided some preliminary estimates about the behavior of oil-stock nexus during COVID-19 pandemic. The results were suggesting that the probability of having negative oil and stock returns during the pandemic may be due to uncertainty associated with the relevant markets. Also, (17) results in study, that daily changes in overall reported cases and total cases of daily deaths induced by COVID-19 have a substantial negative impact on stock returns. With the negative impact of COVID-19 on stock returns becoming more prominent as the study uses total cases of deaths to proxy the effect of this infectious disease.

A recent review of the literature on this topic, the Brent price curve remains in contagion phases attack, where long-term oil was more expensive than short (18). So, the price difference between contracts for delivery now and in 6 months remains in contagion (19). However, it has narrowed significantly over the last 2 months. For the OPEC +, the ideal scenario is to move the curve shape from the current contagion, when comparing prices are lower than future prices (14, 20), into a mild backwardation, with spot prices higher than forward ones. OPEC is still focusing on increasing revenues sustainably through a combination of higher prices and a more significant market share (21).

Research has tended to focus on COVID-19 Cases rather than COVID-19 deaths. An additional problem is now opinion presents a new approach to gives an insight into the relationship between oil prices and the number of deaths caused by Coronavirus. Also, the rapid spread of COVID-19 deaths triggers shockwaves on both the stock market and the oil crude, as well, in the real economy. Then, the depth of the new economic downturn will impact on the policy response to the coronavirus crisis through economic, traffic, trade, financial, and public behavior prevention (22, 23).

Arouri and Fouquau (24) and Arouri et al. 2012 (25) which reviewed the long-term relation between GCC stock markets and oil prices, also came to the opposite conclusion. Surprisingly, the two studies showed that positive data shows that there is a robust long-term correlation in all countries except Saudi Arabia (is negative).

Nevertheless, Albulescu (26) believes Coronavirus (COVID-19) creates fear and uncertainty, hitting the global economy and amplifying the volatility of the financial market. The study investigates the impact of COVID-19 numbers on crude oil prices while controlling for the impact of financial volatility and the United States (US) economic policy uncertainty. The finding shows that the new cases and new deaths have a marginally negative impact on the crude oil prices in the long run.

Concerns have arisen, the increase in the death rate for COVID-19 causes more stagnation and less production, travel, and commercial ties, which explains the decrease in oil prices (27, 28). That describes the countries implementation of large-scale prevention policy, and an attempt to revitalize the economy, through the budget and job support, allowing for a slight recovery in the market supported by preventive measures (28, 29).

According to Matuka (30) study, the COVID-19 outbreak is causing shock waves in financial markets and the real economy around the World, and a policy reaction is depending on the depth of the future recession. The study discusses the effect on the uncertainty of the US economic strategy of COVID-19 (measured by the number of new cases and deaths) and oil prices. The study results suggest that new cases in the USA have a substantial impact on the market. However, there are no significant impacts on economic policy uncertainty in death cases. Also, there is a reverse relationship between branch oil prices and political uncertainty which can boost economic policy uncertainty as branch oil prices decline. A similar relationship observed between the number of cases and the VIX index (31).

The empirical evidence concerning the correlation between oil prices and the disease of Coronavirus, and the explanations that affected they are positive (32) investigated the impact of the COVID-19 pandemic on the Chinese stock market. The prevalence of COVID-19 has measured by the daily growth in total confirmed cases and the daily growth in total deaths from COVID-19. The results of their estimate show that the reduction in both daily growth in total confirmed cases and total deaths from COVID-19 involves Increase equity returns across all companies. They also demonstrate that the control variables are negative and meaningful.

Also, Al-Marri et al. (33) used the Swamy-Arora method to analyze panel data to examine the Tunisian stock market's reaction to the current COVID-19 pandemic. COVID-19 measured by the daily growth of confirmed cases, the daily growth of the death toll and the daily growth of recovered cases. They concluded that the daily growth of confirmed cases has a positive relationship with inventory returns, while the daily growth of mortality cases adversely affects stock returns performance. On the contrary, the daily growth of recovered cases has a positive but not significant effect. At the same time, they support that the Tunisian authorities have an essential role to play in combating the spread of the epidemic by taking early preventive measures to protect the population and save the economy.

Also, using the daily change in the main stock index and the daily growth in confirmed COVID-19 cases and death data from 64 countries, (34) confirms that stock markets are responding negatively to the increase in confirmed COVID-19 cases. It also documents a weak stock market response to the growth in the number of deaths due to COVID-19.

Overall, the effect on China's stock market returns and the uncertainty of the COVID-19 pandemic. (34) results of the study indicate that changes daily in overall reported cases of COVID-19 in China, as total deaths and as daily cases, have significant negative impacts in inventory returns with a more pronounced negative effect on inventory returns through COVID-19. Deaths decrease the impact of this contagious epidemic. The findings further show that COVID-19 has a favorable effect on the volatility of these stock returns and has convergent validity. We have recently seen that infectious diseases, such as COVID-19, may have a significant effect on the stock yield and uncertainty. The findings are essential to consider the effect on the Chinese stock market of COVID-19.

Saudi Arabia supported the stability of high oil price levels and is seeking to take steps to help open rapidly to the outside World (35, 36), and its implications for prices. The effects of a delayed investment in China and Europe will eventually help economic recovery by only managing the epidemic and reducing mortality rates. Provided that the impact of more favorable monetary policies and targeted government fiscal responses generates a recovery, so oil prices are likely to hit new reasonable levels and medium-term rehab (29, 37, 38).

Widely considered to be the most important, COVID-19 spreads human suffering around the World. At the same time, the pandemic in KSA is less advanced, and the economic effect is significant due to its large amounts of arrivals and strong precautionary measures in comparison with other countries.

The next decade is likely to see OPEC and Saudi Arabia are confronted with daunting tasks to challenge group unity. If OPEC is to manage effectively this decade, the quality of research, strategy, and decision-making must be significantly improved in light of coronavirus deaths cases increasing.

Also, many experts now contend hypotheses regarding COVID-19 deaths appear to be ill-defined debatable. There is a significant negative relationship between the confirmed cases and death cases from COVID-19 and the volume trading on the stock exchange in KSA. In the short term, it will be challenging to adapt supply to the possibly faltering demand for recovery because of the recovery of output from non-OPEC (oil prices stability). Despite this interest, no one, to the best of our knowledge, examined the nature of the impact that correlates coronavirus deaths cases with crude oil prices in the Kingdom of Saudi Arabia and its economy, and COVID-19 spillover using ARDL specification.

## Materials and Methods

### Data and Variables

This study explores the current economic situation and the potential effects of COVID-19 death cases on the economy of the Kingdom of Saudi Arabia, based on trends critical financial market indicators of crude oil prices. The involves an assessment of the possible economic impacts and necessary activities (recommendations and findings).

The study data consisted of a daily time series of oil prices in Saudi Arabia (See: https://oilprice.com/oil-price-charts), and the development of COVID 19 cases in the World and the United States of America (See: https://ourworldindata.org/coronavirus-source-data), from the beginning of the Corona pandemic (January 22, 2020) to (June 14, 2020).

Hence, the study variables were dependent variable daily oil prices in Saudi Arabia, independent variables: death (the number of deaths in the World), and deaths_USA (the number of deaths in the United States of America).

### ARDL Models

The data of the study, using an econometric model, what has called the Autoregressive-Distributed Lag (ARDL) models, which introduced by Pesaran and Shin (39) and developed by Pesaran et al. (40) to examine the relationship between death cases and oil prices in Saudi Arabia, according to the following equation: See (41).

$$\begin{array}{l} PP_{\text{t}}=\beta_{0}+\overset{\text{k}}{\underset{\text{i}=1}{\sum }}\beta_{\text{i}}*PP_{\text{t}-\text{i}}+\overset{\text{k}}{\underset{\text{i}=1}{\sum }}\gamma_{\text{i}}*Death_{\text{t}-\text{i}}+\overset{\text{k}}{\underset{\text{i}=1}{\sum }}\alpha_{\text{i}}Death_{\text{t}-\text{i}} \end{array}$$

After assessing the model, several tests performed, the most important of which was the test of the significant of the parameters. Then every time we deleted the non-significant parameters. Finally, we came up with the optimal model that achieves the significance of all parameters and assumptions regarding residuals (42), according to the automatic selection method (Gets) General to specifics methodology (43). All this done automatically using OxMetrics.

## Oil Prices and Coronavirus Cases

### Oil Prices in Saudi Arabia

**Figure 2:**
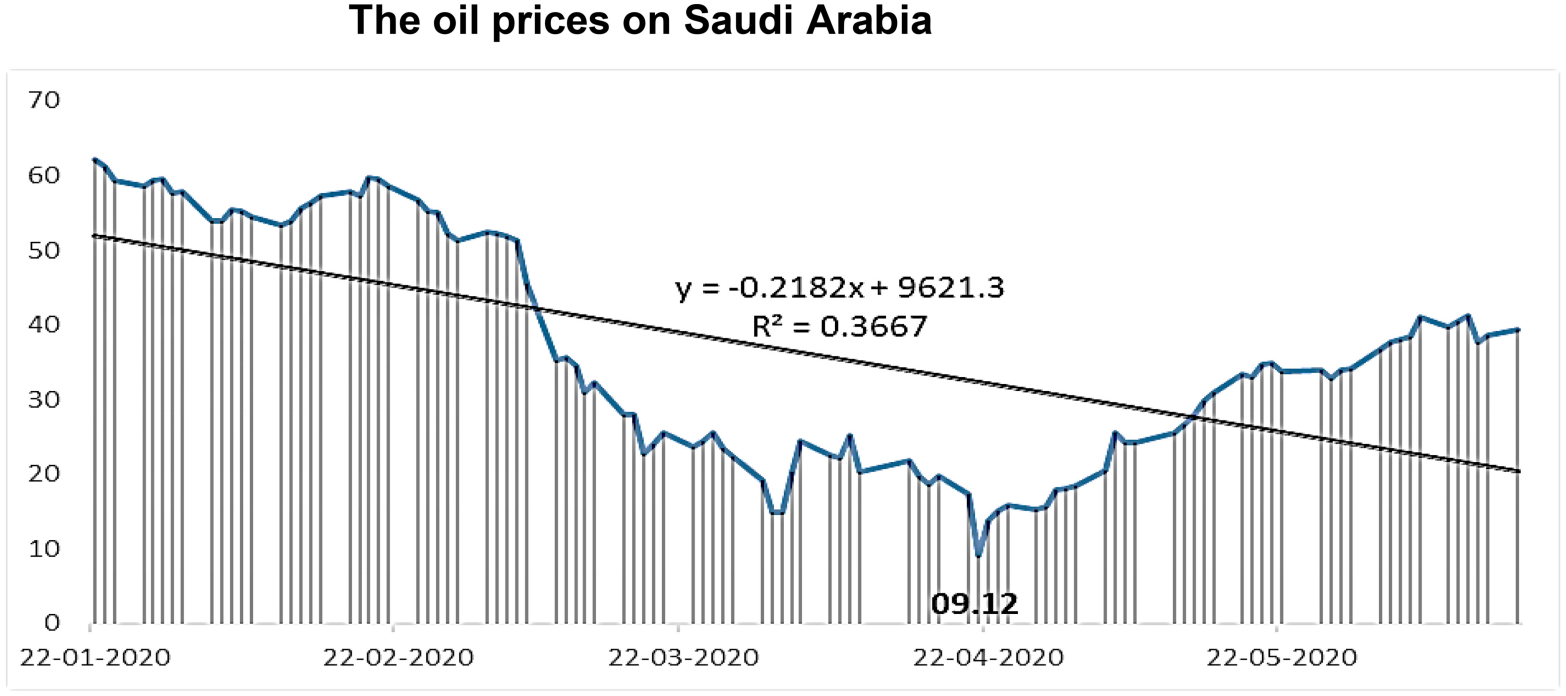
The oil prices on Saudi Arabia. Source: Authors' calculations based on data from oil-price-charts.

The Figure 2. indicates exactly that oil prices are constantly decreasing according to the equation (y = −0.2182x + 9621.3), until they reached their minimum level (9.12 dollars per barrel) on April 21 2020 and then increased again until they reached (39.44 dollars per barrel. There is evidence to suggest the negative impact of the Corona pandemic on oil prices in Saudi Arabia and other countries (44). It is very probable OPEC decision to cut supply until the market regains balance, as prior inquiries centered on OPEC behavior.

Kaufmann (45) and Frondel and Horvath (46) they studied OPEC's effect on oil prices and found that OPEC supply quotas and OPEC's capacity to offset export capacities to maintain medium to long market stability.

### Coronavirus: Cases and Deaths

**Figure 3:**
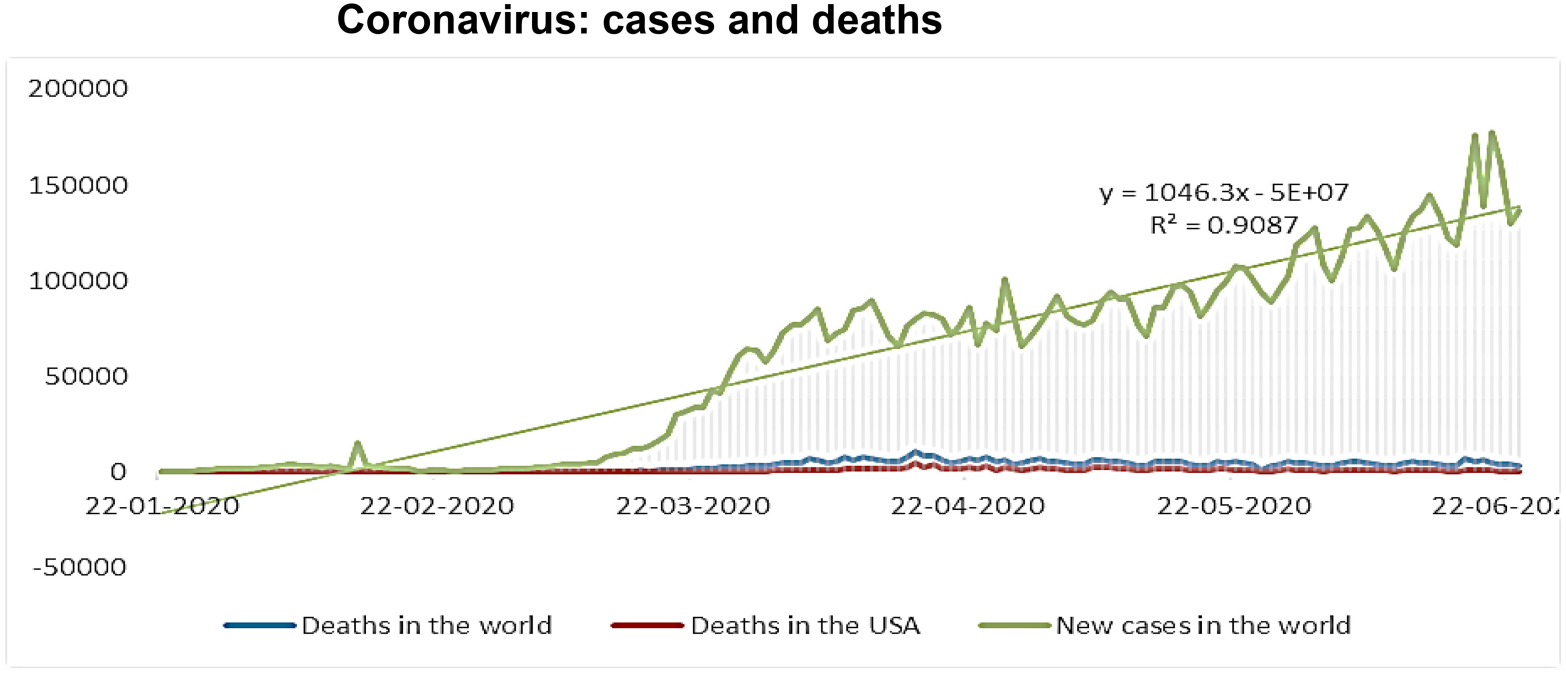
Coronavirus: cases and deaths. Source: Authors' calculations, based on who.int data.

Figure 3 illustrates that the cases of Coronavirus are continually increasing according to the equation (y = 1046.3x −5E+07). In contrast, the deaths in the World after April 16, 2020, where they arrived (10,520 cases in the World) have begun to decline. Thus, confirms the beginning and knowledge of different countries of the World how to deal with patients with HIV (47). See more detail: WHO, Situation Report −164, July 2, 2020 (48).

Table 1 demonstrates the mean oil prices in the study period reached (35.99), and this indicates a decline in oil prices due to the Corona pandemic, despite their variation in the minimum value (9.12 $ per barrel) and the maximum value (62.11 $ per barrel) and explained by High standard deviation (15.02). However, during the study time, Coronavirus deaths mean 3,021 deaths in the World and only 26 deaths in the USA. With the standard deviation that reflects the apparent dispersion of coronavirus deaths, a period of record-high rates (8,468 deaths in the World and 84 deaths in the USA), and times decrease (0 deaths in the World and the USA) with the onset of the pandemic.

**Table 1:** Descriptive statistics.

**Normality test for oil prices**	
Observations	145
Mean	35.991
Std. Devn.	15.021
Skewness	0.28134
Excess Kurtosis	−1.3263
Minimum	9.1200
Maximum	62.110
Median	33.875
Madn	19.518
Asymptotic test: Chi^∧^2 (2) = 12.540 (0.0019)^**^	
Normality test: Chi^∧^2 (2) = 30.773 (0.0000)^**^	
**Normality test for death in KSA**	
Observations	145
Mean	3021.1
Std. Devn.	2616.0
Skewness	0.15682
Excess Kurtosis	−1.3995
Minimum	0.00000
Maximum	8468.0
Median	3413.0
Madn	4047.5
Asymptotic test: Chi^∧^2 (2) = 12.428 (0.0020)^**^	
Normality test: Chi^∧^2 (2) = 27.721 (0.0000)^**^	
**Normality test for death in USA**	
Observations	145
Mean	26.050
Std. Devn.	22.296
Skewness	0.56192
Excess Kurtosis	−0.54419
Minimum	0.00000
Maximum	84.210
Median	21.660
Madn	30.527
Asymptotic test: Chi^∧^2 (2) = 12.610 (0.0018)^**^	
Normality test: Chi^∧^2 (2) = 28.234 (0.0000)^**^	

** *Significant at 0.01*.

This apparent dispersion in both oil prices and corona deaths requires a robust econometric model (the ARDL model).

## Empirical Results

Using the automatic selection method according to the Gets (General to specifics) methodology, we entered the ARDL model (7.7.7). After a comparison of dozens of models, we've obtained the ARDL (1.4.4) model that achieves parameter significance and strong characterization according to the tests used, as shown Table 2.

**Table 2:** The ARDL model tests.

	**Coefficient**	**Str. error**	***t*-value**	***t*-prob**	**Part.R^**∧**^2**
OP	0.849330	0.02918	29.1	0.0000	0.8696
Canstant	8.23057	1.636	5.03	0.0000	0.1661
Death KSA	−0.000370447	0.0001150	−3.22	0.0016	0.0756
Death USA	−0.0738713	0.01261	−5.86	0.0000	0.2128
Sigma	1.59361	RSS		322.52902	
R^∧^2	0.988629		*F*_(3, 127)_ = 3,681 (0.000) ^**^		
Adj. R^∧^2	0.988361	Log—likelihood	−244. 896		
No. of observations	131	No. of parameters		4	
Mean (OP)	34.5298	Se (OP)		14.7713	
**1- step (ex post) forecast analysis 2020-06-08 - 2020-06-14**					
**Parameter constancy forecast tests:**					
**Forecast Chi**^**∧**^**2 (7)** = 6.2776 (0.5077)					
**Chow** ***F***_**(7, 127)**_ = 0.88364 (0.5215)					
**AR 1 - 2 test:** *F*_(2, 125)_ = 0.42427 (0.6552)					
**ARCH 1-1 test:** *F*_(1, 129)_ = 1.5791 (0.2112)					
**Normality test:** Chi^∧^2(2) = 38.228 (0.0000)^**^					
**Hetero test:** *F*_(6, 124)_ = 2.4359 (0.0292)*					
**Hetero-X test:** *F*_(9, 121)_ = 1.7695 (0.0808)					
**RESET23 test:** *F*_(2, 125)_ = 0.12899 (0.8791)					

** *Significant at 0.01*.

### ARDL Model Test

It is apparent from Table 2 the significance of all parameters (according to t-prob, which are <0.05) as the effect coefficient was 0.84 concerning oil prices for the previous period, −0.00037 about death KSA, and −0.073 with death USA. The coefficient of determination was 0.9886, which means the high explanatory power of the model. All other descriptive studies have shown good model results.

It can see in It is also in the World and the United States of America that oil prices in Saudi Arabia are adversely affected by deaths (prices began to rise as mortality rates fell) (37). It explains why Saudi Arabia's economy linked to the financial market trends and oil policy and vice versa. The ARDL model (1.4.4) can write in the following formula:

$$\begin{array}{l} PP_{t}=8.23+0.84*PP_{\text{t}-1}-0.0037*{Death\;KSA}_{\text{t}-4}\\ \;-0.073*{Death\;USA}_{\text{t}-4} \end{array}$$

**Figure 4:**
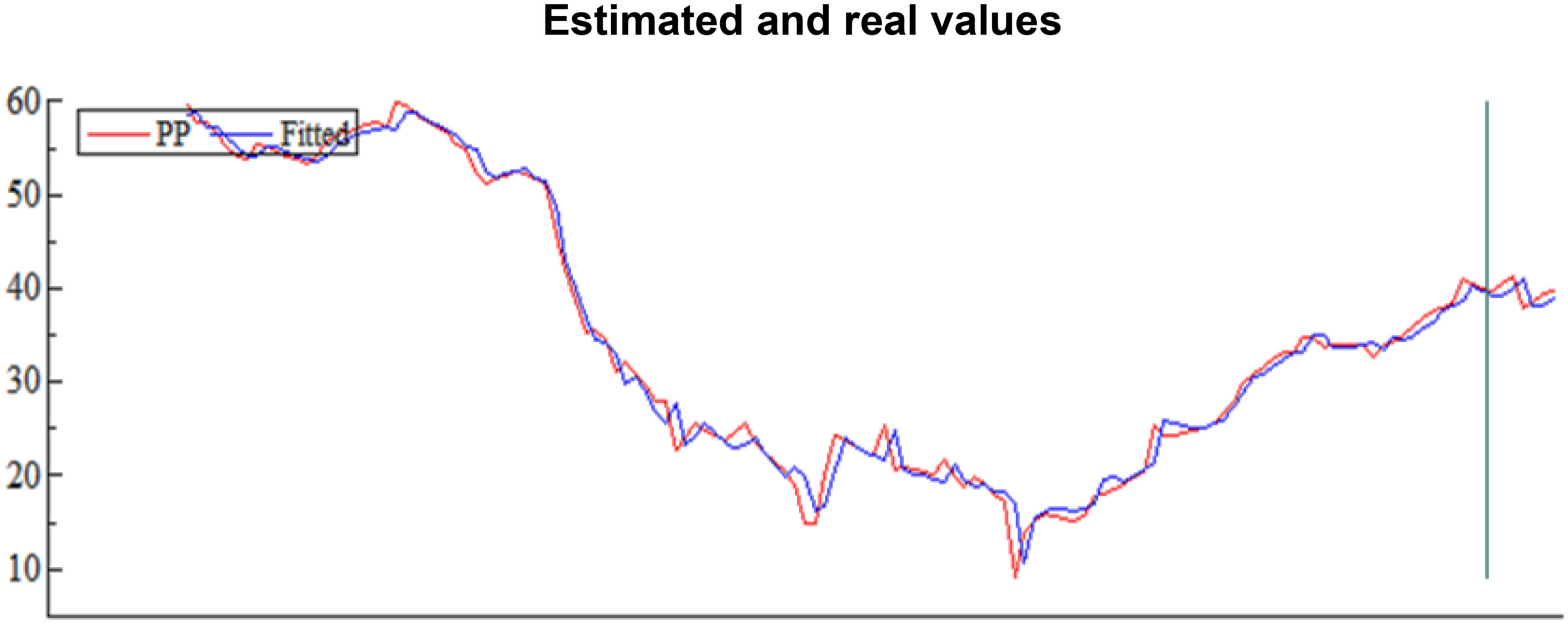
Estimated and real values.

For the estimated period, it was from January 22 to June 7; we kept seven observations to compare the predictive value with the real value, as shown in Figure 4.

Histogram, we can see a broad match between the estimated values and the real values, as evidenced by the chow test in Table 2; this confirms the validity of the ARDL model that links deaths in the World as independent variables and oil prices in Saudi Arabia as dependent variables.

### Modeling and Dynamic Analysis

The table below represents the long-term effects.

Table 3. highlights that the impact factor of death rates in the United States −0.49 was much more significant than the impact factor of the number of deaths in the World −0.0024, which reflects the impact of the United States and its economy on changes in oil prices in Saudi Arabia (49, 50).

**Table 3:** The long-term effects.

	**Coefficient**	**Std. Error**	***t*-value**	***t*-prob**
Solved static long-run equation for OP				
Canstant	54.6265	1.658	33.0	0.0000
Death KSA	−0.00245866	0.0004081	−6.02	0.0000
Death USA	−0.490285	0.04763	−10.3	0.0000
Long-run sigma = 10.5768				

### Shock Analysis

The following figure shows the long and negative impact of a shock on the independent variables, and how this effect begins to decrease until it fades after about 30 days.

**Figure 5:**
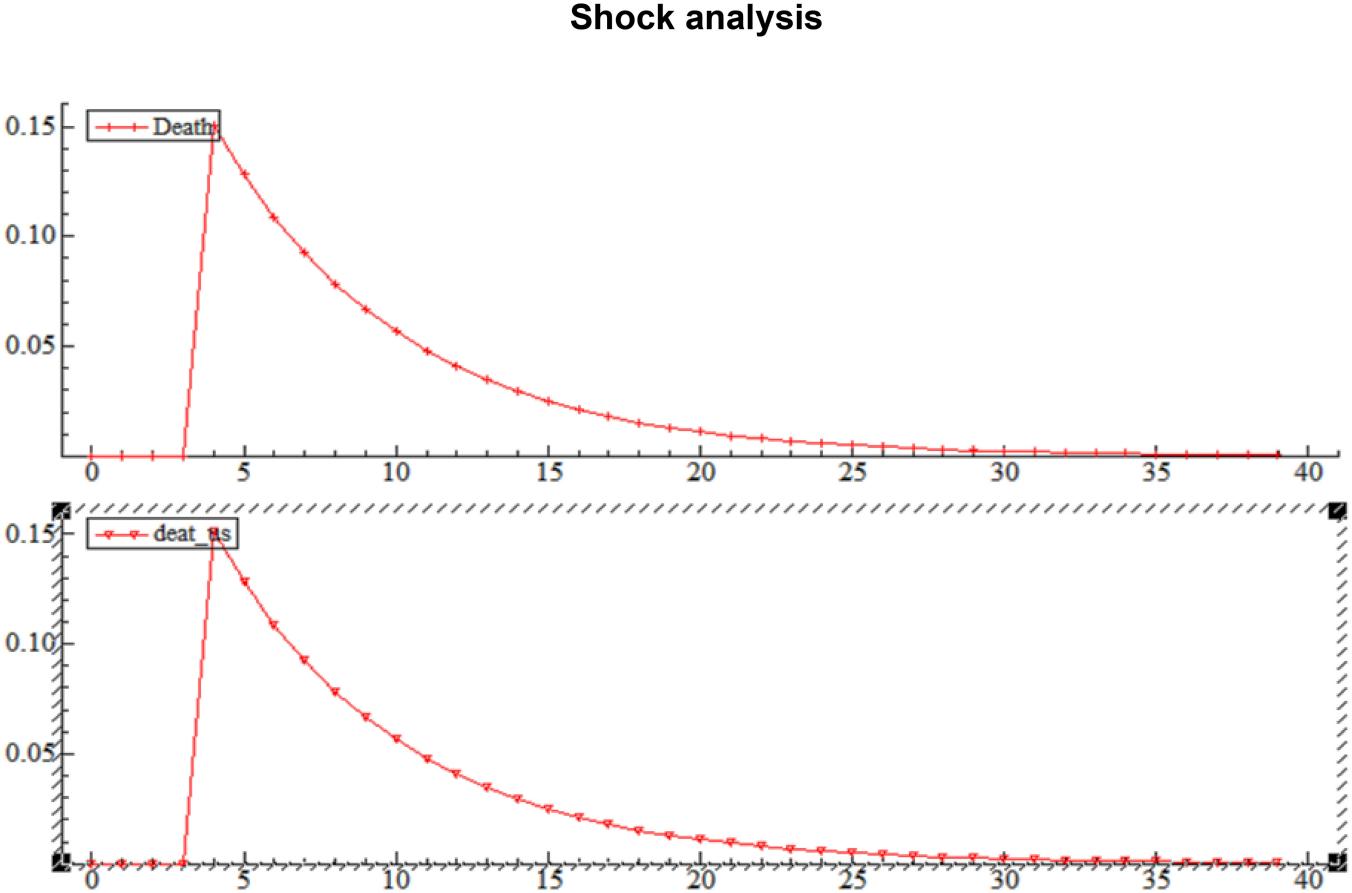
Shock analysis.

Figure 5 shows a clear trend of high uncertainty of death between March 12 and April 9; the period has been characterizing by a very rapid global spread of the virus, followed by a significant rise in deaths in Italy, especially France, Spain, and the USA. The volatility curve of the Crude Oil Index indicates high volatility between July 24 and August 14.

## Discussion of Results

With the spread of the COVID-19 epidemic, growth, travel, and trade stagnated, and the price of oil decreases. So, it explains the interest conflict of debt producers with different budget requirements, fiscal deficits, and market expectations, this substantiates previous findings in the literature.

There has been some disagreement concerning Saudi Arabia hopes to keep oil prices high, and Russia seeks to achieve a share of world oil markets and their impact on prices. Moreover, later spending in China and Europe, once the epidemic is under control, will ultimately promote economic recovery, since the effect of better monetary policy and targeted fiscal responses from governments will lead to recovery. As such, oil prices are likely to reach new lower levels, but a medium-term recovery is in sight.

We have found that investors changed their investment strategy as oil spot prices reported a first decline that contributed to high volatility in the future commodity market during the first cycle of coronavirus spread. Our results are consistent with a theory of future expectations on the market, which shows that speculators can predict future price patterns as economic agents (51).

We believe that the quick spread of COVID-19 deaths causes a shock wave of the economy, oil, and real estate of the financial market and the depth of the economic downturn depends on the political response to the crises of the Coronavirus by opening up the markets, investment, trade, industrial growth, and public prevention. The excess oil stocks would weigh the oil price in the short term; strict OPEC discipline will need to clear these stocks over the next few years, faced with a slow rebound in demand (52) has provided some preliminary estimates about the behavior of oil-stock nexus during COVID-19 pandemic. The results were suggesting that the probability of having negative oil and stock returns during the pandemic, maybe due to uncertainty associated with the relevant markets. Unexpected situations such as a pandemic can have a significant effect on market fundamentals in the short term, and there have been correlations with indexes and oil.

Our study provides additional support, OPEC must also handle the recovery of crude oil output as demand and oil prices increase. The analysis calls for the two possibilities to involve OPEC Crude Oil. In the middle of 2020, in the negative case, the demand for OPEC crude oil increased to 32 MMbpd in 2024 and then fell to 31 MMbpd. The market for OPEC oil growth in the 2030s, following secular declines of several major non-OPEC producers, especially Russia. The plant remarkably counts from the main basins for the two Covid-19 scenario models in the spring of 2020, the delays due to both falling and rising oil prices.

This result not anticipated, the reason for this is probably, after the Covid-19 pandemic, the widening gap between oil demand and supply in the US, triggered by the high death rate in the mid-2020s. The fundamental explanation expected for this is the decline in US production from 2020 to 2024. In the early 2020s, net oil imports projected to hit 3–4 MMbpd. Coronavirus has a significant effect on the global economy, and thus oil demand, in particular in the most likely cause of a deep and sustained recession linked to a prolonged pandemic. Until mid-2023, the average crude oil price will remain below $50/bbl without active OPEC action.

The results indicate that COVID-19 deaths toll has a significant impact on oil prices. However, it mainly influences the situation reported in the United States. If we evaluate the case outside the United States, we can see the positive impact on oil prices of the estimates of the COVID-19 death. Hence the amplification of mortality risks in the stock market and the real economy caused by the increasing economic instability in the United States.

The evidence from this study suggests the impact of the pandemic will surely reach oil prices, which will continue to decline, at least in the short term, as a result of lower demand for crude oil. Here comes the role of OPEC, a central element in reducing oil price losses through the consent of OPEC members. The reduction in market production thus promotes crude oil prices and improves cash resources.

Our work has led us to conclude OPEC and Saudi Arabia are confronted with daunting tasks to challenge group unity. If OPEC is to manage effectively this decade, the quality of research, strategy, and decision-making must be significantly improved. In the short term, it will be challenging to adapt supply to the possibly faltering demand for recovery because of the recovery of output from non-OPEC.

## Conclusion

The study focuses on modeling the effects of the COVID-19 deaths on oil prices in KSA during the period from January 22, 2020, to June 14, 2020, using an ARDL estimation approach. The oil prices of the USA is the endogenous variable while COVID-19 daily deaths case is the Corona prevalence's measure. Previous researches about the effects of the COVID-19 deaths epidemic on oil prices limited, while this study is a contribution to this recently emerging literature.

The results indicate the substantial effect of COVID-19 death numbers on the oil price. However, the situation in the United States has significantly affected these findings. When we analyzed the case outside the US, it founded that COVID-19 death numbers have a positive impact on the price of oil. We are therefore stressing a rise in the risk of deaths on the financial market and on the real economy attributable to a growing economic instability caused by a policy in the United States.

Our research suggests that the policymakers should encourage there is an urgent need to develop prudent policies describing the magnitude of the COVID-19 epidemic and potential social and economic devastation, Because of the severe human, societal, and economic consequences. We think that our findings could provide an overview of the possible effects of a pandemic on the oil market. COVID-19 will lead to increasingly shattered losses (10). Intense multispectral efforts needed for determining the potential economic impacts of an outbreak, to explain how the economy affects different outbreak scenarios, rather than providing the best predictions for the epidemic scale (7). So that the World of GDP growth expected to slow, as, despite the negative impact on COVID−19, the Organization for Economic Co-operation and Development predicts a global recession as growth remains positive in all areas of the economy (9, 53, 54). Gherghina examined the linkages in financial markets during coronavirus disease 2019 (COVID-19) pandemic outbreak for the following economies: USA, Spain, Italy, France, Germany, UK, China, and Romania. The quantitative approach reveals a negative effect of the new deaths' cases from Italy on the 10-year Romanian bond yield both in the short-run and long-run (55).

Further work needs to done many economies have already adopted absolute policies, and many nations, in particular the United States of America, have reduced interest rates, continued the 2019 facilitation round, and have taken financial support steps (56).

The aim is to direct policies to calculate the risk of a pandemic so that prevention and rapid response costs can adequately assess. The worst-case scenario is expected in the event of a rise in viral infection in the countries of the region, the effect of lower oil demand, the rise in OPEC supply and severe coronavirus consequences.

## Data Availability Statement

Publicly available datasets were analyzed in this study. This data can be found at: https://ourworldindata.org/coronavirus-source-data~https://oilprice.com/oil-price-charts.

## Author Contributions

All authors listed have made a substantial, direct and intellectual contribution to the work, and approved it for publication.

## Conflict of Interest

The authors declare that the research was conducted in the absence of any commercial or financial relationships that could be construed as a potential conflict of interest.
